# Dietary supplementation of cysteamine promotes *Hu* lamb growth performance via somatotropic axis potentiation

**DOI:** 10.5713/ab.250803

**Published:** 2026-06-24

**Authors:** Qinwu Li, Tongtong Xie, Xuan Wang, Fadi Li, Fei Li, Tao Guo, Long Guo

**Affiliations:** 1 State Key Laboratory of Grassland Agro-ecosystems, Key Laboratory of Grassland Livestock Industry Innovation, Ministry of Agriculture and Rural Affairs, Engineering Research Center of Grassland Industry, Ministry of Education, College of Pastoral Agriculture Science and Technology, Lanzhou University, Lanzhou, China

**Keywords:** Cysteamine, Growth Performance, *Hu* Lamb, mTOR Signaling Pathway, Muscle Development

## Abstract

**Objective:**

Cysteamine (CS) is known for its ability to regulate hormone secretion. Although widely used as a growth promoter for ruminants, long-term administration at high doses results in intestinal ulceration and growth retardation. We have previously demonstrated that long-term supplementing 20 mg CS/kg body weight (BW) does not affect growth performance.

**Methods:**

This study aimed to elucidate the impacts of 30 mg CS kg^−1^ BW administered for 30 days on growth performance, hormonal profiles, rumen fermentation parameters, rumen bacterial communities, and muscle characteristics in *Hu* lambs. This dosage and duration regimen was effective in promoting growth performance while avoiding the ruminal negative effects observed at higher doses. Thirty-six male *Hu* lambs were randomly assigned to two groups.

**Results:**

The CS supplementation exhibited increased final BW, average daily gain, and dry matter intake during the first 21 days of the experimental period (p<0.05). Hormonal analysis revealed that CS supplementation increased the levels of growth hormone, luteinizing hormone in the pituitary, testosterone (T) in the testis, and insulin-like growth factor I in the serumwhile decreasing the levels of somatostatin in the hypothalamus and serum (p<0.05). Rumen fermentation analysis indicated an increase in total volatile fatty acids and the proportion of valerate (p<0.05), while the proportion of acetate and the acetate/propionate ratio decreased (p<0.05). Muscle analysis revealed decreased mRNA expression levels of *MyHCIIb* (p<0.05) in the *longissimus dorsi* muscle. Western blot analysis showed increased expression of mTOR proteins, along with an elevated phosphorylation level of mTOR (p<0.05) in the *longissimus dorsi* muscle.

**Conclusion:**

Collectively, this study demonstrates that dietary CS supplementation enhances growth performance in *Hu* lambs by improving rumen function and modulating somatotropic axis hormones.

## INTRODUCTION

Growth hormone (GH) is a crucial regulator of growth rates in mammals, present in various tissues and organs, with its release primarily controlled by growth hormone-releasing hormone (GHRH) [1]. GH has been demonstrated to enhance production performance in ruminants through multiple pathways [2]. However, the direct administration of GH to animals has been prohibited in many countries due to concerns regarding potential risks to animal health and food safety [3]. An alternative approach to improve production performance involves stimulating endogenous GH production. Somatostatin (SS), a hormone that inhibits GH secretion by antagonizing GHRH, plays a key role in regulating GH levels [1]. Reducing SS production can effectively enhance GH secretion, thereby increasing insulin-like growth factor I (IGF-I) levels and improving growth and production efficiency in animals.

Cysteamine (CS; HSCH_2_CH_2_NH_2_) is a bioactive thiol compound derived from coenzyme A (CoA) that has shown potential to enhance production performance in various livestock species when administered either orally or intravenously [4]. Current mechanistic insights indicate that CS primarily enhances growth performance by affecting the somatotropic axis [5], which regulates SS secretion and subsequently increases GH concentrations [6].

Studies have shown that low-dose CS (10–20 mg kg^−1^ body weight [BW]) fails to improve average daily gain (ADG) or final BW in lambs [7]. Consistent with that, our 80-day trial at 20 mg kg^−1^ BW confirmed no benefits for growth, digestibility, or rumen fermentation [8]. Doubling the dose to 40–80 mg kg^−1^ BW increases ADG in sheep [9,10], but intestinal ulceration emerges when prolonged high-dose CS is administered [11–13]. Therefore, in fattening lambs, it is worth studying whether the CS addition can improve the growth performance while avoiding mucosal damage and dry matter intake (DMI) decrease. This study specifically tested a single dosage of CS at 30 mg/kg BW administered for 30 days, a regimen demonstrating efficacy in promoting growth performance while avoiding the ruminal adverse effects observed at higher doses.

While the growth-promoting effects of CS through SS antagonism and subsequent GH release are well-established in monogastric animals, the mechanisms by which CS supplementation influences in rumen microflora and muscle development in ruminants remain unclear. Hormones regulated by CS, such as IGF-I and testosterone (T), have been shown to enhance bone and muscle quality [14] and promote the growth of type I muscle fibers, as well as increase muscle fiber cross-sectional area [15]. These processes involve the JAK-STAT and PI3K-AKT-mTOR signaling pathways [16,17]. Although CS has been demonstrated to elevate circulating levels of IGF-1 and T in *Hu* lambs, its downstream effects on mTOR-mediated muscle protein synthesis and myofiber type specification remain unquantified. Therefore, the present study aims to explore the effects and mechanisms of dietary CS supplementation on growth performance, rumen function, and the *longissimus dorsi* phenotype of *Hu* lambs.

## MATERIALS AND METHODS

### Animals, experimental design, and diets

Thirty-six male *Hu* lambs (4 months of age; initial BW 34.08± 0.47 kg) were recruited and randomly allocated to two treatment groups (n = 18/group) using a completely randomized design: (A) a basic diet (CON), and (B) a basic diet with 30 mg/kg BW CS. Under identical conditions, a previous study showed that 20 mg CS/kg BW exerted negligible effects on *Hu* lamb performance [8]. The CS used in this study was composite metal CS chelate from Changsha Green Top Biotech (CN111096393A, ≥50% CS). The basic diet was formulated based on NRC [18] and made into pellet total mixed ration (P-TMR). Feed composition and nutritional levels are shown in Table 1.

A total of 18 *Hu* lambs per treatment were used for growth performance evaluation. At the end of the 30-day experimental period, lambs were hierarchically randomized for subsequent analyses: 10 lambs per treatment were selected for slaughter performance; and among these, 8 lambs were further selected for rumen fluid collection and digestibility determination. All randomizations were performed using a random number generator, and initial BWs were balanced across subgroups (p>0.05).

Six lambs were finally selected from the aforementioned 8 for integrated tissue collection and subsequent hormone and molecular analyses, with 2 excluded due to limited tissue yield or postmortem tissue damage to ensure consistent sample quality across all tissue-level endpoints.

All lambs were housed in individual pens equipped with individual feed troughs and water nipples, allowing precise monitoring of individual feed intake throughout the experiment. Lambs were fed twice daily at 08:00 and 18:00. After a 14-day adaptation period (fed the basal diet), lambs were randomly assigned to experimental treatments and group-fed for a 30-day experimental period, consisting of a 21-day growth performance test followed by a 9-day digestibility trial. The CS was administered at 30 mg/kg BW daily, calculated based on individual BW recorded weekly. The calculated CS dose was accurately weighed and thoroughly mixed with the morning feed portion (1/2 of daily ration) to ensure complete consumption. Feed troughs were inspected visually 30 min after feeding to confirm complete consumption of the CS-mixed feed; Any refused feed was collected, weighed, and recorded; if refusal occurred, the CS dose was recalculated and re-administered with the subsequent feeding after adjusting for the refused amount. No CS refusals were observed during the experimental period, indicating complete consumption of the administered dose.

### Feed intake and production

A total of 18 *Hu* lambs per treatment were used for growth performance evaluation. All experimental lambs were weighed before feeding on the morning of day 1 and day 21 of the experimental period, recorded as initial and final BWs, and ADG was calculated for the 21-day period. Feed offered and refused were recorded daily to determine DMI and feed-to-gain ratio (F/G).

### Measurement of nutrient apparent digestibility

From day 22 to day 30, apparent nutrient digestibility was determined using eight *Hu* lambs per treatment. The digestion trial consisted of a 3-day adaptation phase (days 22–24) followed by a 6-day total fecal collection phase (days 25–30). Fecal samples were collected directly from the rectum five times daily during the collection phase. The analysis of DM, OM, and CP was performed [19].

The content of both NDF and ADF in the diet and feces was determined using heat-stable α-amylase and sodium sulphite, based on the method described by Van Soest et al. [20]. Acid insoluble ash (AIA; internal marker, recovery >96%) concentrations in diet and feces were measured. Total apparent digestibility (TAD) was calculated as:


(1) TAD (%)=100-(A/B)×(C/D)×100

where A = AIA concentration in the diet; B = AIA concentration in feces; C = nutrient concentration in feces; and D = nutrient concentration in the diet.

### Slaughter characteristics

At the end of the 30-day experimental period, ten lambs per treatment were fasted for 12 hours and weighed to obtain live weight at slaughter. Lambs were then slaughtered by jugular venipuncture exsanguination. Carcass weight was recorded, and dressing percentage was calculated. Eight of the 10 slaughtered lambs were digestibility experiment.

### Hormone assays

Blood samples were collected from six *Hu* lambs per treatment. before slaughter, allowed to clot at 25°C for 2 hours, and centrifuged at 3,000×g for 5 minutes to obtain serum and stored at −20°C until analysis. Hypothalamic, pituitary, and testicular tissues were harvested immediately after slaughter, rinsed with ice-cold PBS, snap-frozen in liquid nitrogen, and stored at −80°C. For GHRH, GH, SS, and luteinizing hormone (LH) determination, frozen tissues were homogenized in ice-cold PBS containing protease inhibitors, followed by centrifugation at 12,000×g for 15 min at 4°C. For T determination, testicular tissues were homogenized directly in the lysis buffer provided in the ELISA kit at a ratio of 1:5 (w/v). The homogenates were centrifuged at 12,000×g for 15 min at 4°C. All supernatants were collected for ELISA analysis.

Determination of GHRH, GH, SS, IGF-I, T, and LH concentrations was conducted using commercially available ELISA kits (Ml025558B, Ml025620B, Ml529742B, Ml025641B, Ml061827B, and Ml025568B, respectively; Shanghai Enzymes Biotechnology) according to the manufacturer’s instructions. The limit of detection (LOD) for GHRH was below 0.1 pg/mL. Intra and inter assay coefficients of variation (CV) were <15% for GHRH, <10% and <15% for GH, SS IGF-I and GnRH, respectively, and <10% for T and LH. All kits were validated for ovine species, with sensitivity specifications confirmed by the manufacturer.

### Hematoxylin-eosin staining

The *longissimus dorsi* muscle samples were cut into about 2 cm^3^ and stored in 4% paraformaldehyde (PFA) from six *Hu* lambs per treatment. After being embedded in paraffin, they were cut into 5 μm slices. After dewaxing, they were stained with hematoxylin and eosin. Images were captured using a microscope (SWE-CX63; Servicebio).

### Ruminal fluid sample collection and analysis

At the end of the slaughter, 8 lambs from digestibility experiment, rumen fluid was collected and filtered through four layers of gauze. Ten milliliters of filtrate were preserved by adding 2 mL of 250 g/L metaphosphoric acid in a 5:1 ratio and stored at −20°C for later volatile fatty acid (VFA) concentration analysis. The VFA analysis was performed using a gas chromatograph (Thermo Fisher Scientific) equipped with a capillary column (30 m×0.32 mm×0.25 μm), following the method described by Li et al. [21]. Additionally, triplicate rumen fluid samples were collected and stored at −80°C for subsequent ruminal microbial DNA extraction [22].

### Microbial DNA extraction, quantitative polymerase chain reaction analysis, and 16S rRNA sequencing

According to instructions, total microbial DNA was extracted from rumen fluid of 8 *Hu* lambs selected from each group using MagMAX-the 96 DNA Multiple Sample Kit (Thermo Fisher Scientific, Cat No.4413021). 16S rDNA sequencing analysis was conducted on the Illumina Novaseq 6000 platform (Baike Biotechnology). High-quality sequences with 97% sequence similarity were clustered and partitioned using operational classification units (OTUs). To investigate the effect of CS addition on rumen bacteria in *Hu* lambs, we evaluated α-diversity and analyzed the differences in microbial community composition using principal coordinate analysis (PCoA). Statistical differences were determined using two-tailed t-tests for α-diversity and PERMANOVA for β-diversity and richness of rumen bacteria of *Hu* lambs in the two groups.

### Gene expression by real-time quantitative polymerase chain reaction

Muscle tissue was snap-frozen, ground under liquid nitrogen, and ~100 mg powder was extracted with 1 mL Accurate Biology RNA reagent. Total RNA (quantified on a NanoDrop One) was reverse-transcribed with the same vendor’s kit. The quantitative polymerase chain reaction (qPCR) was run in triplicate on a CFX96 with Accurate Biology master mix, gene-specific primers (Oligo 7-designed, AuGCT-synthesized; Supplement 1), GAPDH as reference, and 2-ΔΔCT analysis.

### Protein expression and modification analysis

Total protein was extracted with a total protein extraction kit (R0010; Solarbio). Relative protein expression level was detected using Western blot after total muscle protein was extracted according to our previous study [23]. After incubation in Enhanced Chemiluminescent Substrate (BL520A; Biosharp) for 1 min, the chemiluminescence of bands was detected using a ChemDoc XRS+ Imaging System (Bio-Rad) and was analyzed with Image J software (National Institutes of Health). Information about antibodies was shown in Supplement 2, and β-tubulin was the internal standard protein.

### Statistical analysis

Growth and slaughter performance, rumen fermentation, and nutrient apparent digestibility data were analyzed using the Statistical Package for the Social Sciences (SPSS) software (ver. 17.0; SPSS). Descriptive statistics, including means and standard errors, were generated. An independent samples t-test was employed to compare group means.

For hormone concentrations in tissue and serum, histomorphology and muscle fiber type of the *longissimus dorsi* muscle, and signaling pathway determination data, GraphPad Prism 10 software (ver. 10.5; GraphPad Software) was used. Data were first assessed for normality using Normality and Lognormality Tests. Values of p<0.05 were considered to indicate statistical difference, and values of 0.05<p<0.1 were considered to indicate a trend of difference.

## RESULTS

### Growth and slaughter performance

As shown in Table 2, the final BW, daily gain, DMI, live weight before slaughter, and carcass weight of *Hu* lambs all increased (p<0.05) with the addition of CS, while the dressing percentage decreased significantly (p<0.05).

### Hormone concentration

Figure 1 shows that with the addition of CS, the levels of GH in the pituitary and IGF-I in the serum of *Hu* lambs increased (p<0.01, Figures 1D, 1G), while the levels of SS in the hypothalamus and serum decreased (p<0.05, Figures 1B, 1E). Notably, hypothalamic GHRH levels in CS group was not different with CON (p>0.05, Figure 1A). Figure 2 shows shat with the addition of CS, the levels of LH in the pituitary and T in the testis of *Hu* lambs increased (p<0.05, Figures 2B, 2C).

### Rumen fermentation and nutrient apparent digestibility

As shown in Table 3, with the addition of CS, the total VFAs and the proportions of valerate in *Hu* lambs increased (p< 0.05), while the proportion of acetate (p<0.01) and the acetate/propionate ratio (p = 0.05) decreased. Meanwhile, Table 4 indicates that there was no significant difference in the apparent digestibility of nutrients between the two treatment groups.

### Compositional profiles and differences of the rumen bacteria

There were no significant differences in the α-diversity between the two groups (Supplement 3B), suggesting similar bacterial evenness. As for PCoA, there was no separation of rumen bacteria between CS group and CON group in *Hu* lambs, indicating CS supplementation had little effect on rumen bacteria of *Hu* lambs. As shown in Supplement 3D, the annotation results of the relative abundance of the top 10 bacterial taxa were analyzed. Uncultured bacteria and species with less than 0.5% abundance in the sample were merged into an “other” category. At the phylum level, the rumen bacterial composition of the two groups was similar, with Bacteroidetes, Firmicutes, and Proteobacteria being the dominant phyla, accounting for more than 90% of the total bacterial community.

### Histomorphology and muscle fiber type of *longissimus dorsi* muscle

Supplement 4 shows that the addition of CS in the diet has no significant effect on the number, cross-sectional area, and fascicular thickness of the *longissimus dorsi* muscle in *Hu* lambs. However, Figure 3 indicates that the addition of CS in the diet affects the expression of *MyHC* gene subtypes in the *longissimus dorsi* muscle. Specifically, CS reduces the mRNA expression level of *MyHCIIb* (p<0.05, Figure 3C), while showing a trend of increasing the mRNA expression levels of *MyHCI*, *MyHCIIa*, and *MyHCIIx* (p<0.10, Figures 3A, 3B, 3D).

### Expression of hormone receptor-related genes in *longissimus dorsi* muscle

Compared with the CON group, the expression level of androgen receptor (AR) mRNA in the *longissimus dorsi* muscle of *Hu* lambs in the CS group showed a decreasing trend (p<0.10, Figure 4C), while the levels of GHR and IGF-IR mRNA remained unchanged (p>0.10, Figures 4A, 4B).

### Expression of JAK2-STAT5 and Akt-mTOR signaling pathway proteins in *longissimus dorsi* muscle

Regarding protein phosphorylation levels, the phosphorylation level of mTOR protein in lambs of the CS group was significantly higher than that in the CON group (p<0.01, Figure 5D). Additionally, the expression levels of STAT5 and mTOR proteins were increased in the CS group compared with the CON group (p<0.05, Supplement 5C and Figure 5E).

## DISCUSSION

In the present study, dietary supplementation of CS at 30 mg/kg BW demonstrated an improvement in the growth performance of *Hu* lambs. The enhancement in ADG may be attributed to both increased feed intake and the inherent growth-promoting properties of CS. Barnett et al. [9] reported that CS can improve ADG independently of changes in DMI, suggesting that CS has direct effects on growth processes. Furthermore, T. Wu et al. [24] found that CS could enhance both DMI and ADG in lambs, which aligns with our findings. These results indicate that CS contributes to growth performance through multiple pathways, including direct physiological effects and increased nutrient intake. Additionally, research has demonstrated that dietary CS influences plasma GH levels in a dose-dependent manner [7,25]. For instance, a dosage of 50 mg/kg BW of CS raises GH levels, while 100 mg/kg BW decreases them, and a dosage of 140 mg/kg BW may lead to gastric ulcers in animals [11,12]. In this study, *Hu* lambs were fed P-TMR containing 30 mg/kg BW of CS. The lamb exhibited improved production performance without any adverse effects on the digestive system. These findings suggest that incorporating CS is an effective strategy for utilizing additives.

The predominant cell type in the pituitary gland is the acidophilic GH secreting cells. Two hypothalamic peptides, GHRH and SS, regulate GH synthesis and secretion from these cells [1]. Consistent with these findings, our study found that CS supplementation decreased SS levels in both the hypothalamus and serum, suggesting that CS exerts an inhibitory effect on SS. This effect is attributed to the thiol group in CS, which acts as a reducing agent and disrupts the disulfide bonds within the SS molecule. Notably, hypothalamic GHRH levels remained unchanged following CS treatment, which is consistent with the known mechanism of CS acting on SS, without directly targeting GHRH or the pituitary [26,27]. Furthermore, the addition of CS resulted in increased GH levels in the pituitary and elevated IGF-I levels in the serum, aligning with previous research. In yaks, GH levels increased by 29% with the administration of 5 g/d of CS over 28 days [28], while in lambs, GH levels rose by 57.42% with 60 mg/kg BW of CS for 64 days [29]. Within the endocrine system, GH and IGF-I primarily govern animal growth performance [30,31]. Overall, this research indicates that CS enhances animal growth performance by modulating hormones within the somatotropic axis.

In this study, dietary supplementation with CS did not affect rumen pH, which remained within the normal range. A previous study demonstrated that CS could reduce the acetate to propionate ratio and increase total VFAs in lambs when administered at a dosage of 60 mg/kg BW for 64 days [29]. This finding is consistent with the results of the current study. The acetate to propionate ratio and the proportion of acetate were lower in the CS treatment group of *Hu* lambs compared to the CON group, while total VFA levels increased. Similar results have been observed in goats [24] and dairy buffalo [32], indicating that the application of CS enhances rumen fermentation efficiency. Additionally, CS has been reported to enhance the production of propionate during rumen fermentation in lambs [29]. Although the proportion of propionate did not differ significantly between the CS and control groups in this experiment, the increase in total VFAs and the decrease in acetate proportion induced by CS still shifted the rumen fermentation towards propionate production. These changes suggest that CS altered the rumen fermentation pattern to enhance the production of propionate in fattening *Hu* lambs, thereby improving energy utilization efficiency. In summary, dietary supplementation with coated CS positively impacted rumen fermentation, likely contributing to enhanced production performance.

The health status and metabolic activities of ruminants are influenced by the diversity of rumen microbiota [33–35]. This study employed 16S rRNA sequencing to analyze the rumen bacterial diversity in *Hu* lambs. The results indicated that CS did not affect the bacterial α-diversity indices or β-diversity in short time. Consistent with previous studies, Bacteroidetes and Firmicutes were identified as the dominant bacterial phyla [24]. These null findings do not exclude the possibility of subtle community changes below the detection threshold of 16S rRNA sequencing, but suggest that any functional effects of CS on rumen metabolism were not accompanied by major taxonomic shifts during the 30-day period. This interpretation aligns with previous studies demonstrating alpha diversity shifts in longer-term supplementation without consistent community-level separation [8], suggesting that intervention duration is a critical factor in elucidating CS and microbiome interactions.

While the value of CS in ruminants has been extensively studied, its effects on muscle in ruminants are less well-documented. Research indicates that GH plays a crucial role in skeletal muscle metabolism, with GH receptors identified in skeletal muscle tissue [36,37]. Given the established effects of CS on hormone secretion in animals, we investigated muscle fiber number, muscle fiber area, and perimysium thickness in the *longissimus dorsi* muscle of *Hu* lambs. However, our findings indicated that the addition of CS did not significantly affect muscle morphological parameters. This outcome contrasts with Bai et al. [38], who found that feeding pigs 70 mg/kg BW of CS increased muscle fiber density. This discrepancy may be attributed to variations in the dosage of CS, as the effect on muscle fiber density could be dose-dependent, diminishing at both low and excessive dosages of CS. The proportion of different muscle fiber types can be indicated by the expression levels of various *MyHC isomers* [39]. Oxidative-slow fibers are rich in *MyHC I* and/or *MyHC IIa*, while glycolytic-fast fibers are characterized by high *MyHC IIb* expression. Additionally, *IIx* fibers represent a metabolic intermediate between *IIa* and *IIb* fibers [40]. Bai et al. [38] observed that supplementing the diet with 70 mg/kg BW of CS reduced the mRNA expression levels of *MyHC IIb* while increasing those of *MyHC I* and *MyHC IIx* in the *longissimus dorsi* muscle of finishing pigs; however, this effect was not evident at a dosage of 140 mg/kg BW of CS. Consistent with these findings, in this experiment, the mRNA expression level of *MyHC IIb* in the *longissimus dorsi* muscle of *Hu* lambs in the CS group decreased compared to the CON group, while the expression levels of *MyHC I*, *MyHC IIa*, and *MyHC IIx* tended to increase. This suggests that dietary supplementation with CS may promote a shift in muscle fiber types from glycolytic-fast to oxidative-slow.

The Hypothalamic-Pituitary-Gonadal Axis is studied to play a significant role in skeletal muscle growth, development, repair, and aging by regulating gonadal hormone secretion [41–43]. In this study, we found that adding CS to the diet decreased AR mRNA expression in the *longissimus dorsi* muscle of *Hu* lambs, while simultaneously increasing LH concentration in pituitary and enhancing mTOR phosphorylation in the *longissimus dorsi* muscle. When T binds to AR, it activates androgen response elements, stimulating the transcription of target proteins and anabolic systems, such as local IGF-I production, which in turn activates the PI3K/Akt/mTOR pathway to enhance protein synthesis [44,45]. In addition to these genomic pathways, T also activates the Akt/mTOR/S6K1 pathway, which is crucial for muscle hypertrophy [46]. Skeletal muscle homeostasis is maintained by balancing protein synthesis and breakdown, with mTOR playing a central role in regulating synthesis [47]. Although direct evidence of the Akt-mTOR pathway’s regulation of muscle fiber types is lacking, existing studies have demonstrated that mTOR can be activated by dietary L-arginine [48] and L-leucine [49] to influence muscle fiber types, highlighting mTOR’s role in determining fiber characteristics. Based on these findings, we speculate that CS may reshape muscle fibers by modulating hormone secretion. Our observations indicate that the addition of CS promotes the transformation of muscle fiber types from glycolytic-fast to oxidative-slow in *Hu* lambs, likely by influencing the secretion of GH and LH from the pituitary. Further research is warranted to elucidate the precise mechanisms underlying these effects and to determine the optimal dosage and timing of CS supplementation for various animal species and physiological conditions.

## CONCLUSION

The results of the present study demonstrate that dietary CS supplementation enhances the growth performance of *Hu* lambs through two primary mechanisms: (1) Modulating the rumen fermentation and increase energy utilization efficiency; and (2) Regulating hormones of the somatotropic axis, which in turn increases pituitary GH secretion and serum IGF-I levels. Furthermore, CS supplementation may indirectly influence muscle fiber characteristics in *Hu* lambs, potentially shifting the balance from glycolytic-fast to oxidative-slow fibers.

## Figures and Tables

**Figure 1:**
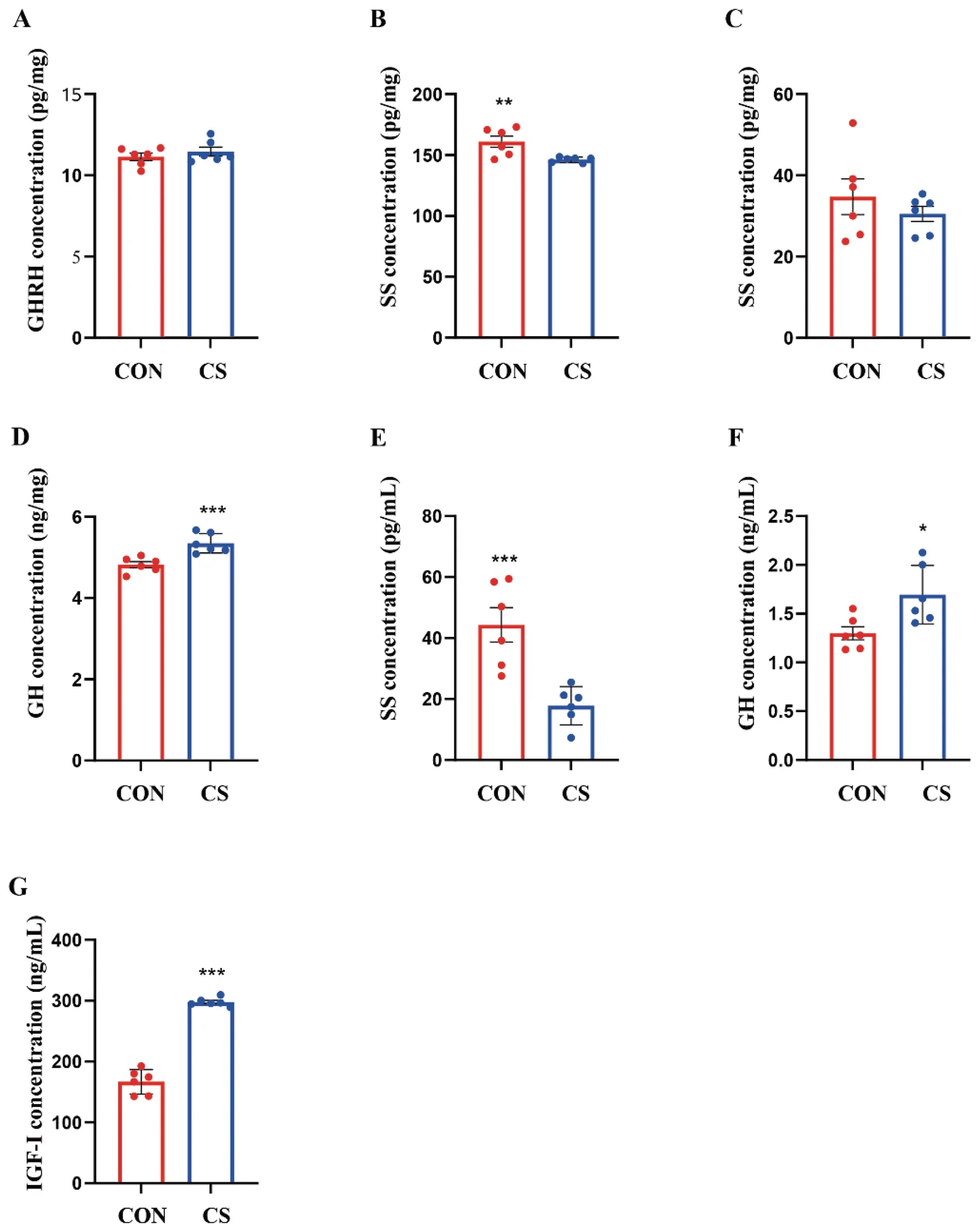
Effects of CS supplementation in diets on somatotropic axis hormone levels of *Hu* lambs. (A) The level of GHRH in the hypothalamus. (B) The level of SS in the hypothalamus. (C) The level of SS in the pituitary. (D) The level of GH in the pituitary. (E) The level of SS in serum. (F) The level of GH in serum. (G) The level of IGF-I in serum. n = 6 per group. Values are the mean±SEM. The red and blue represent the value of each sample, and the height of the bar represents the average value of each group. * 0.05<p<0.10, ** p<0.05, *** p<0.01. GHRH, growth hormone-releasing hormone; CS, cysteamine; SS, somatostatin; GH, growth hormone; IGF-I, insulin-like growth factor I; SEM, standard error of the mean.

**Figure 2:**
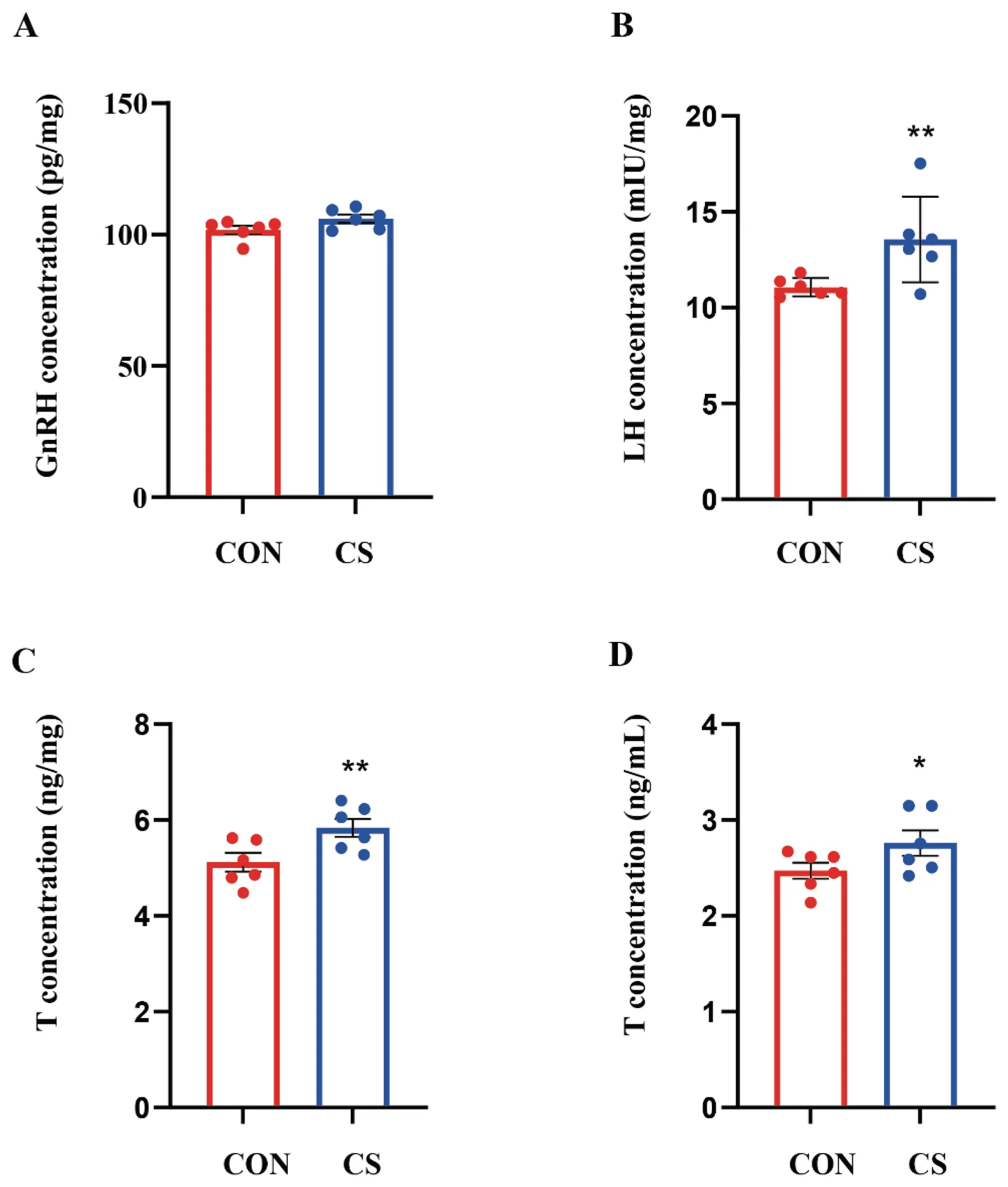
Effects of CS supplementation in diets on gonadal axis hormone levels of *Hu* sheep. (A) The level of GnRH in the hypothalamus. (B) The level of LH in the pituitary. (C) The level of T in the testis. (D) The level of T in serum. n = 6 per group. Values are the mean±SEM. The red and blue represent the value of each sample, and the height of the bar represents the average value of each group. * 0.05<p<0.10, ** p<0.05. GnRH, gonadotropin-releasing hormone; CS, cysteamine; LH, luteinizing hormone; T, testosterone; SEM, standard error of the mean.

**Figure 3:**
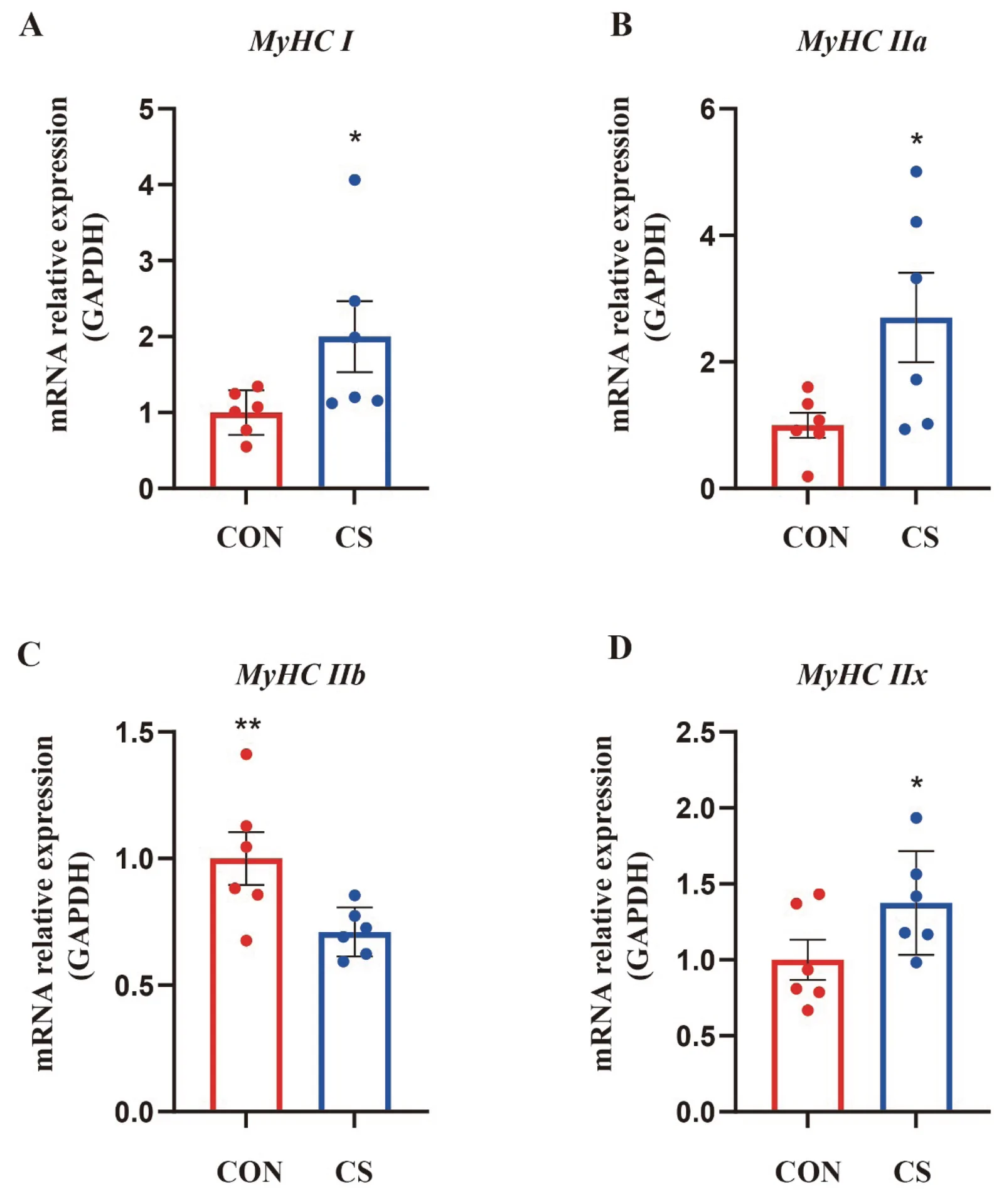
Effects of cysteamine (CS) supplementation in diets on the mRNA expression of *MyHC* gene subtypes of *longissimus dorsi* muscle of *Hu* lambs. (A) mRNA relative expression of *MyHC I*. (B) mRNA relative expression of *MyHC IIa*. (C) mRNA relative expression of *MyHC IIb*. (D) mRNA relative expression of *MyHC IIx*. n = 6 per group. Values are the mean±SEM. The red and blue represent the value of each sample, and the height of the bar represents the average value of each group. * 0.05<p<0.10, ** p<0.05. SEM, standard error of the mean.

**Figure 4:**
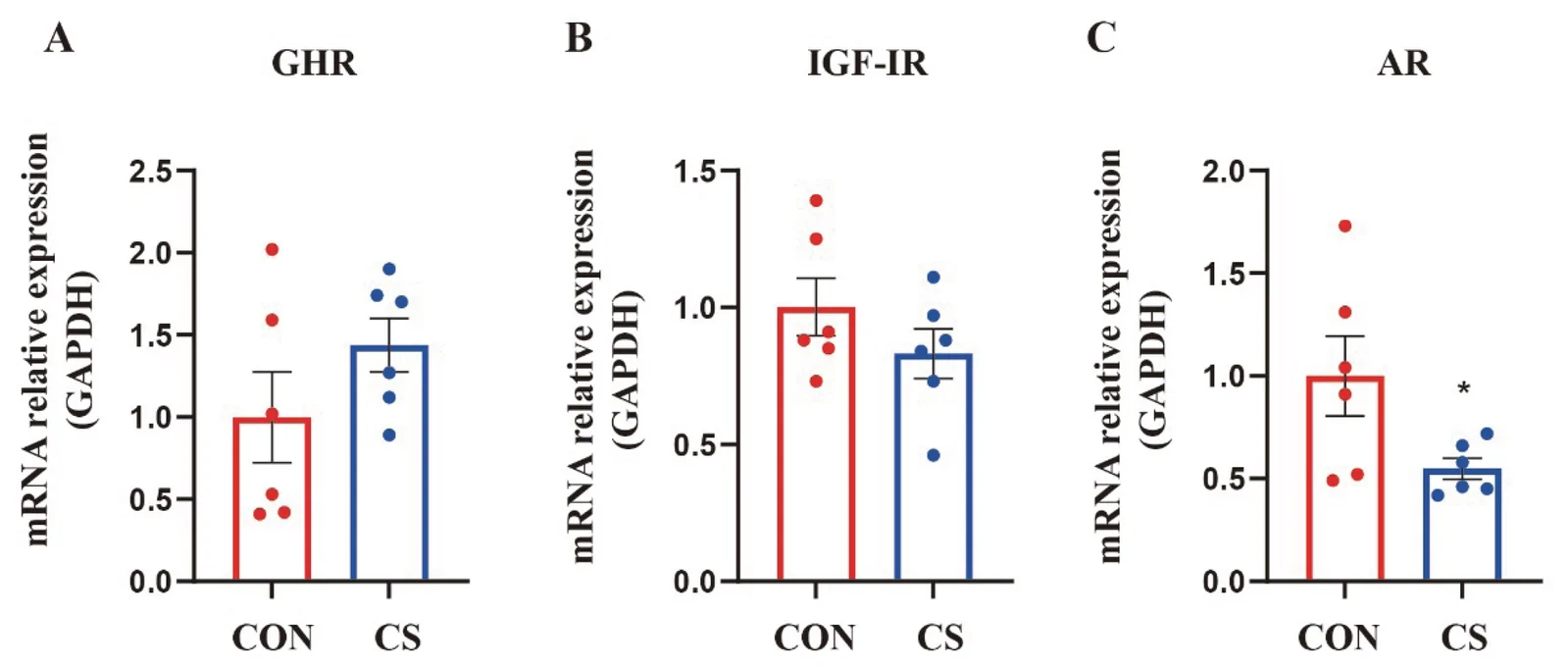
Effects of cysteamine supplementation in diets on mRNA expression of hormone receptor in *longissimus dorsi* muscle of *Hu* lambs. (A) mRNA relative expression of GHR. (B) mRNA relative expression of IGF-IR. (C) mRNA relative expression of AR. n = 6 per group. Values are the mean±SEM. The red and blue represent the value of each sample, and the height of the bar represents the average value of each group. * 0.05<p<0.10. GHR, growth hormone receptor; IGF-I, insulin-like growth factor I receptor; AR, androgen receptor; SEM, standard error of the mean.

**Figure 5:**
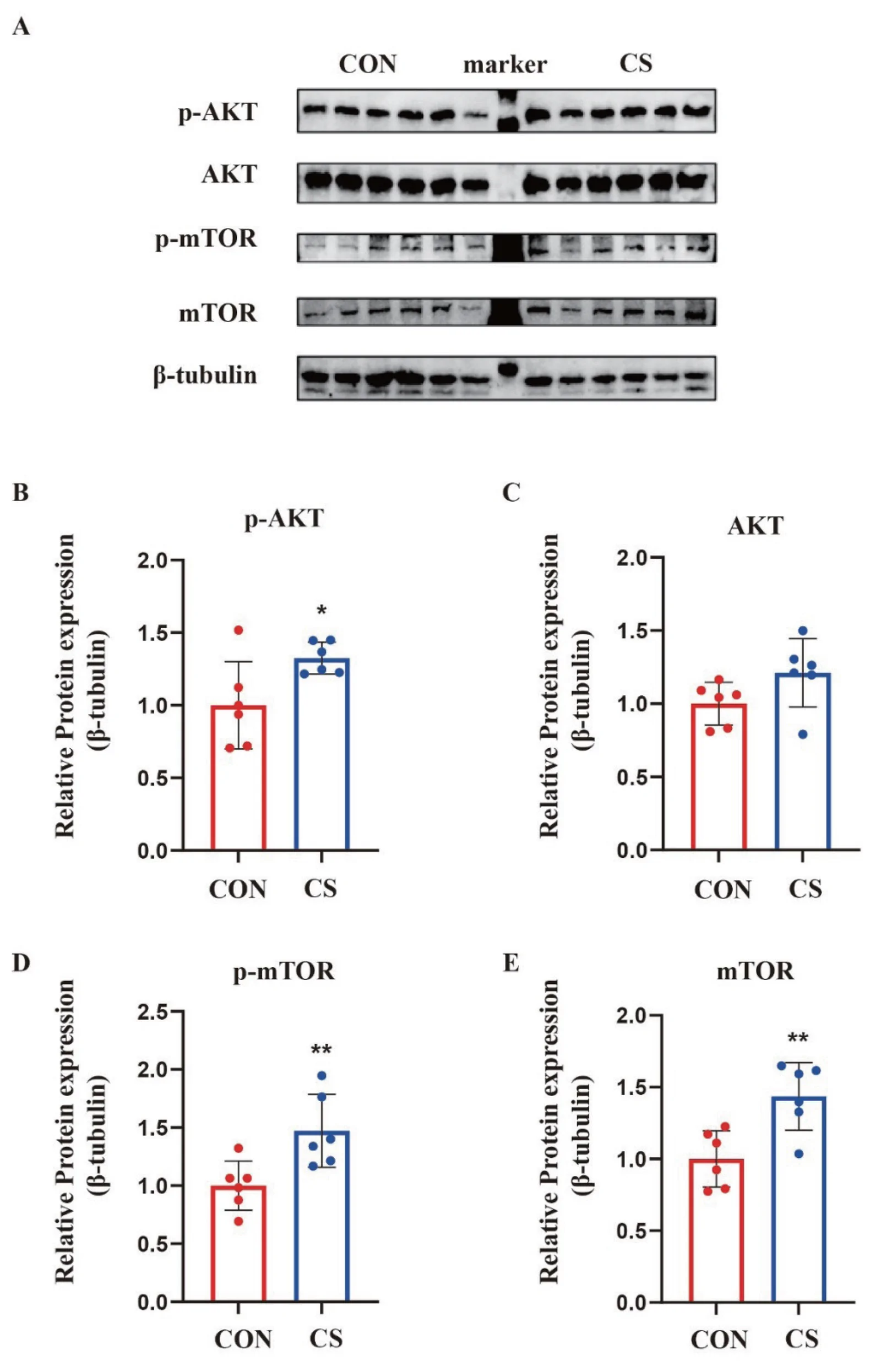
Effects of cysteamine (CS) supplementation in diets on protein expression level of Akt-mTOR signaling pathway in *longissimus dorsi* muscle of *Hu* lambs. (A) The chemiluminescence of bands detected by a ChemDoc XRS+ imaging system. The relative protein expression of (B) p-AKT, (C) AKT, (D) p-mTOR, (E) mTOR against the reference protein β-tubulin. n = 6 per group. Values are the mean±SEM. The red and blue represent the value of each sample, and the height of the bar represents the average value of each group. * 0.05<p<0.1, ** p<0.05. SEM, standard error of the mean.

**Table 1:** Composition and nutrient levels of diet (% of DM)

Item	Diet
Ingredients	
Corn stover	20.00
Corn grain	20.00
Barley	10.00
Molasses	4.00
Expand soybean	3.00
Cottonseed meal	7.00
Corn germ meal	12.00
Soybean hull	10.50
Corn bran	10.00
Limestone	1.20
NaCl	0.50
Expand urea	0.30
NaHCO_3_	1.00
Premix^1)^	0.50
Total	100.00
Nutrient levels	
Dry matter	92.53
Organic matter	92.73
Neutral detergent fiber	35.75
Acid detergent fiber	14.12
Crude protein	14.33

1) The premix provided the following per kilogram of diets: Fe 25 mg, Mn 40 mg, Zn 40 mg, Cu 8 mg, I 0.3 mg, Se 0.2 mg, Co 0.1 mg, VA 20,000 IU, VD3 4,500 IU, VE 20 IU.

**Table 2:** Effects of cysteamine supplementation in diets on growth and slaughter performance of *Hu* lambs

Items	Treatments		SEM	p-value

	CON	CS		
Growth performance				
Initial body weight (kg)	33.39	34.78	0.91	0.14
Final body weight (kg)	39.00^b^	41.62^a^	0.82	0.01
ADG (kg/d)	0.27^b^	0.33^a^	0.02	0.02
DMI (kg/d)	1.50^b^	1.68^a^	0.07	0.02
F/G	6.42	5.57	0.65	0.21
Slaughter performance				
Live weight before slaughter (kg)	41.04^b^	43.93^a^	0.72	<0.01
Carcass weight (kg)	21.12^b^	22.16^a^	0.41	0.02
Dressing percentage (%)	51.47^a^	50.45^b^	0.45	0.04

Growth performance, n = 18 per group; slaughter performance, n = 10 per group.

a,b Means with different superscripts within the same row differ significantly (p<0.05).

CS, cysteamine; SEM, standard error of the mean; ADG, average daily weight gain; DMI, dry matter intake; F/G, feed: gain ratio.

**Table 3:** Effects of cysteamine supplementation in diets on rumen fermentation parameters of *Hu* lambs

Items	Treatments		SEM	p-value

	CON	CS		
pH	6.74	6.62	0.10	0.23
Total VFAs	56.25^b^	67.14^a^	2.41	0.02
Acetate (%)	64.89^a^	59.79^b^	0.94	<0.01
Propionate (%)	22.84	25.53	0.95	0.16
Isobutyrate (%)	1.05	1.10	0.07	0.75
Butyrate (%)	8.31	9.27	0.54	0.39
Isovalerate (%)	1.37	2.16	0.24	0.10
Valerate (%)	1.54^b^	2.15^a^	0.13	0.02
Acetate/propionate	2.88^a^	2.44^b^	0.11	0.05

Fermentation parameters, n = 8 per group.

a,b Means with different superscripts within the same row differ significantly (p<0.05).

CS, cysteamine; SEM, standard error of the mean; VFA, volatile fatty acid.

**Table 4:** Effects of cysteamine supplementation in diets on the nutrient apparent digestibility of *Hu* lambs

Items	Treatments		SEM	p-value

	CON	CS		
DM (%)	63.91	60.88	1.15	0.20
CP (%)	70.56	68.75	1.58	0.58
OM (%)	67.17	64.08	1.14	0.18
NDF (%)	45.49	41.42	1.62	0.22
ADF (%)	41.34	40.20	1.50	0.72

Nutrient apparent digestibility, n = 8 per group.

a,b Means with different superscripts within the same row differ significantly (p<0.05).

CS, cysteamine; SEM, standard error of the mean; DM, dry matter; CP, crude protein; OM, organic matter; NDF, neutral detergent fiber; ADF, acid detergent fiber.

## Data Availability

Upon reasonable request, the datasets of this study can be available from the corresponding author.

## SUPPLEMENTARY MATERIAL

**Supplement 1**. The primers used in the present study.

**Supplement 2**. Antibody information for western blot.

**Supplement 3**. Effects of cysteamine supplementation in diets on rumen bacteria of *Hu* lambs.

**Supplement 4**. Effects of cysteamine supplementation in diets on histological characteristics of *longissimus dorsi* muscle of *Hu* sheep (HE staining).

**Supplement 5**. Effects of cysteamine supplementation in diets on protein expression level of JAK2-STAT5 signaling pathway in *longissimus dorsi* muscle of *Hu* lambs.
