# Supplementary material for: Dietary supplementation of cysteamine promotes Hu lamb growth performance via somatotropic axis potentiation
Source: Anim Biosci. 2026 Jun 24;39(10):250803. doi: 10.5713/ab.250803 (PMC13662936; doi:10.5713/ab.250803)
Supplement: Supplementary file 1 [file ab-250803-Supplementary-1.pdf]

Supplement 1. The primers used in the present study.

Genes	Primer sequences	Accession No.
MyHC I	F: AAGAACCTGCTGCGGCTG<br>R: CCAAGATGTGGCACGGCT	AB058898.1
MyHC IIa	F: GAGGAACAATCCAATACAAATCTATCT<br>R: CCCATAGCATCAGGACACGA	AB058896.1
MyHC IIx	F: GGAGGAACAATCCAATGTCAAC<br>R: GTCACTTTTTAGCATTTGGATGAGTTA	AB058897.1
MyHC IIb	F: GACAACTCCTCTCGCTTTGG<br>R: GGACTGTGATCTCCCCTTGA	XM_027974883.2
GHR	F: GGAGGAAACCATACGAAAATCCA<br>R: AAAGCTGGTGTGGCTTCACT	DQ062717
IGF-1R	F: CTGTGCGAGAAGACGACCAT<br>R: GCGCTGGGACACATTTTCTG	NC_019475.2
AR	F: GGTGAGCAGAGTGCCCTATC<br>R: GTGGTCTCCAAACGCATGTC	KF227907.1
GADPH	F: CACCCTCAAGATTGTCAGC<br>R: CAGTGGTCATAAGTCCCTCC	NM_001190390.1
