# Supplementary material for: Dietary supplementation of cysteamine promotes Hu lamb growth performance via somatotropic axis potentiation
Source: Anim Biosci. 2026 Jun 24;39(10):250803. doi: 10.5713/ab.250803 (PMC13662936; doi:10.5713/ab.250803)
Supplement: Supplementary file 2 [file ab-250803-Supplementary-2.pdf]

Supplement 2. Antibody information for western blot.

Antibody	Category	Source	INC	Cat No.
mTOR	Primary	Rabbit	Cell Signaling Technology	2972
mTOR (Ser2448)	Primary	Rabbit	Cell Signaling Technology	2971
JAK2	Primary	Rabbit	Bioss	bs-23003R
JAK2 (Tyr1007+Tyr1008)	Primary	Rabbit	Bioss	bs-2485R
STAT5	Primary	Rabbit	Abcam	ab194898
STAT5 (Y694)	Primary	Rabbit	Abcam	ab32364
AKT	Primary	Rabbit	Abcam	ab179463
AKT (Ser473)	Primary	Rabbit	Cell Signaling Technology	4060
β-tubulin	Primary	Rabbit	Proteintech	10068-1-AP
Goat Anti-Rabbit IgG	Secondary	Goat	Bioss	bs-0295G
