# Supplementary material for: Dietary supplementation of cysteamine promotes Hu lamb growth performance via somatotropic axis potentiation
Source: Anim Biosci. 2026 Jun 24;39(10):250803. doi: 10.5713/ab.250803 (PMC13662936; doi:10.5713/ab.250803)
Supplement: Supplementary file 3 [file ab-250803-Supplementary-3.pdf]

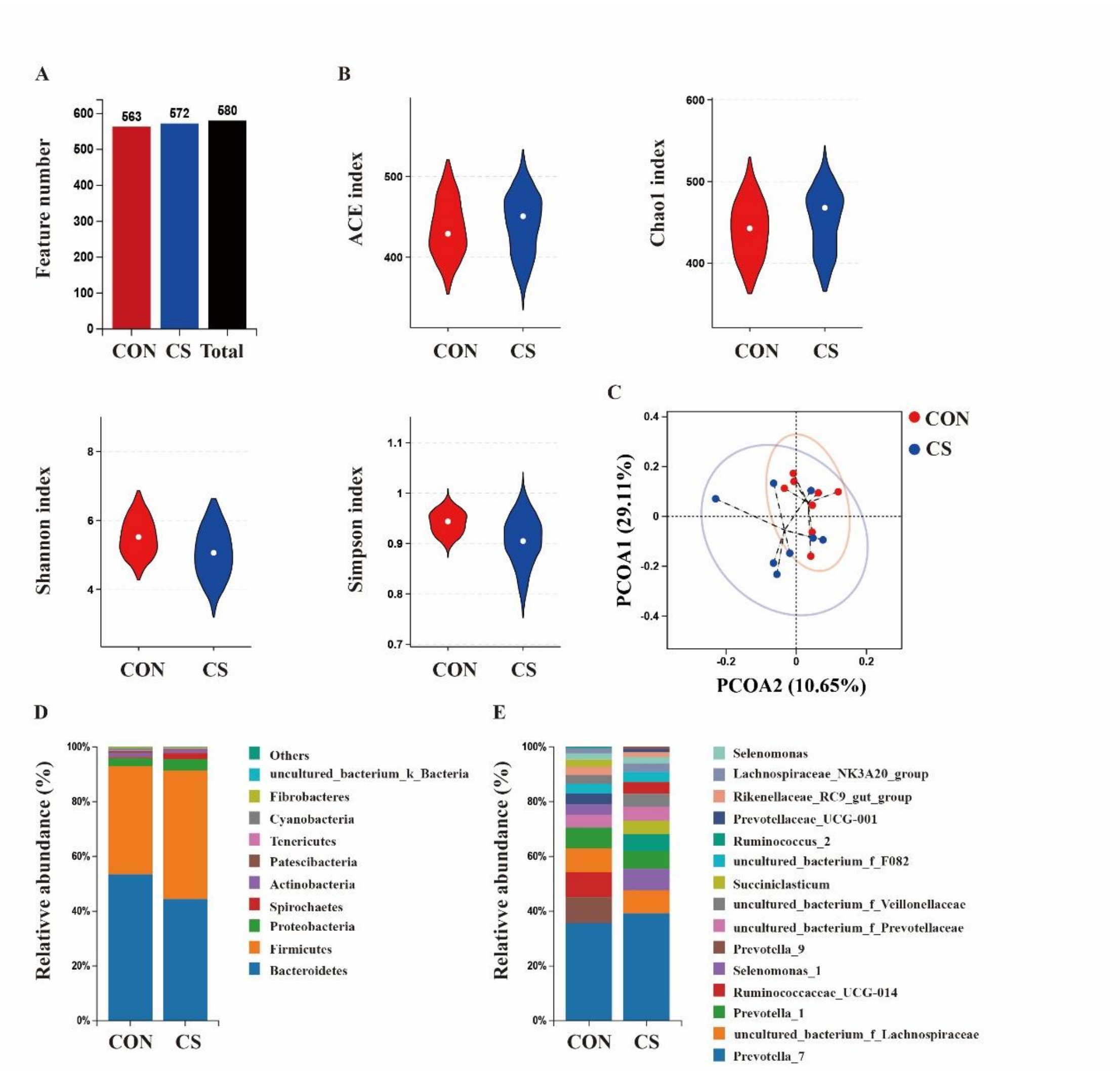

Supplement 3. Effects of cysteamine supplementation in diets on rumen bacteria of *Hu* lambs. (A) Rumen bacterial OTUs. (B) The bacterial α-diversity and (C) β-diversity in the rumen fluid. (D) The composition of rumen bacteria at the phylum level in the TOP 10. (E) The composition of rumen bacteria at the genus level in the TOP 15. Each group contained 8 *Hu* lambs fed either a CON or CS (cysteamine) diet. Values are the mean ± SEM. The red and blue represent the value of each sample, and the height of the bar represents the average value of each group. **$p < 0.05$.
