# Supplementary material for: Dietary supplementation of cysteamine promotes Hu lamb growth performance via somatotropic axis potentiation
Source: Anim Biosci. 2026 Jun 24;39(10):250803. doi: 10.5713/ab.250803 (PMC13662936; doi:10.5713/ab.250803)
Supplement: Supplementary file 4 [file ab-250803-Supplementary-4.pdf]

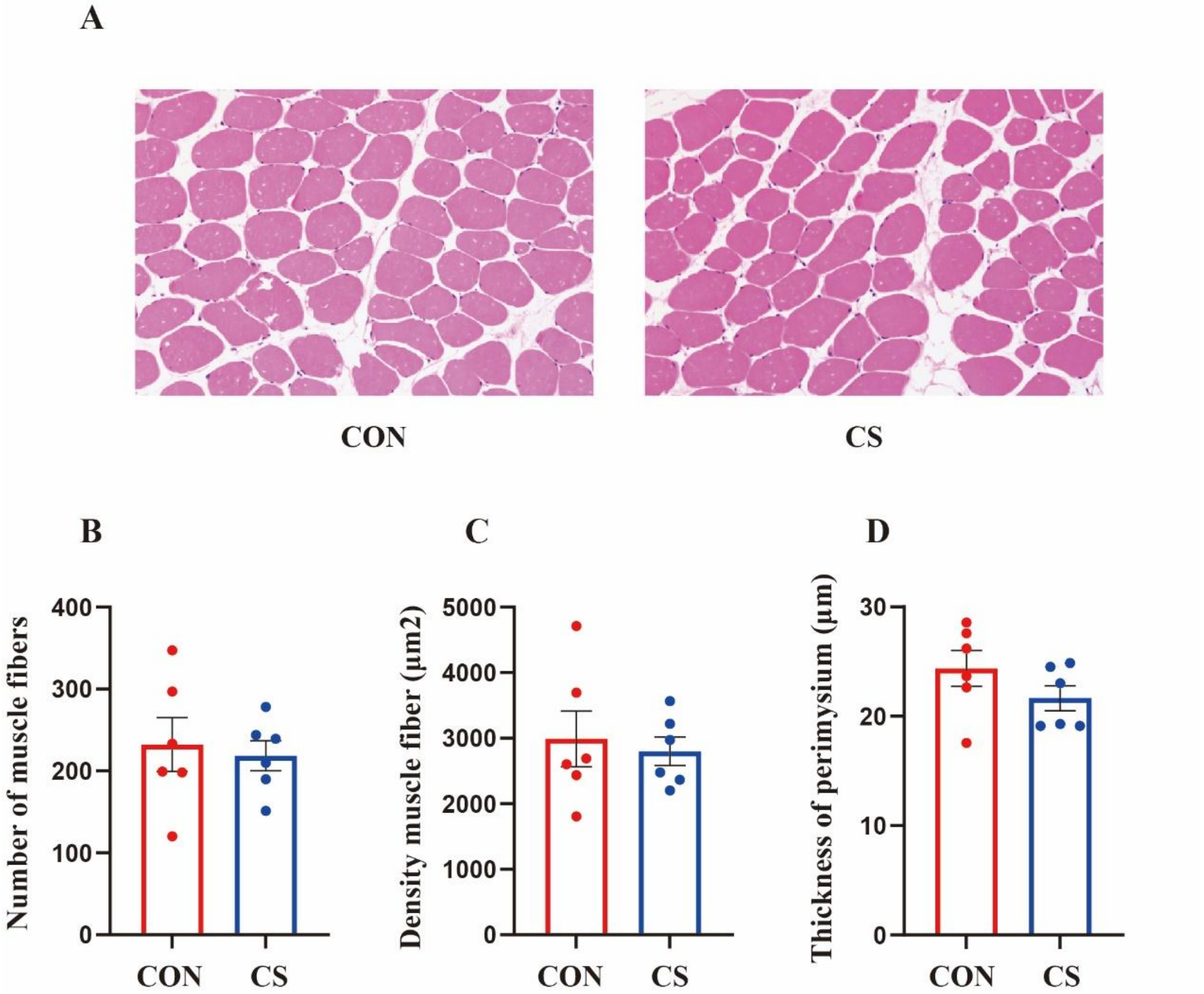

Supplement 4. Effects of cysteamine supplementation in diets on histological characteristics of *longissimus dorsi* muscle of *Hu* sheep (HE staining). (A) HE staining of the tissue sections of the *longissimus dorsi* muscle of Hu sheep. (B) The number of muscle fibers. (C) The density of muscle fibers. (D) The muscle perimysium thickness of lambs. Each group contained 6 *Hu* lambs fed either a CON or CS (cysteamine) diet. Values are the mean ±SEM. The red and blue represent the value of each sample, and the height of the bar represents the average value of each group. (Magnification, 40×, bar = 10 μm). *$0.05 < p < 0.10$, **$p < 0.05$.
