# Supplementary material for: Dietary supplementation of cysteamine promotes Hu lamb growth performance via somatotropic axis potentiation
Source: Anim Biosci. 2026 Jun 24;39(10):250803. doi: 10.5713/ab.250803 (PMC13662936; doi:10.5713/ab.250803)
Supplement: Supplementary file 5 [file ab-250803-Supplementary-5.pdf]

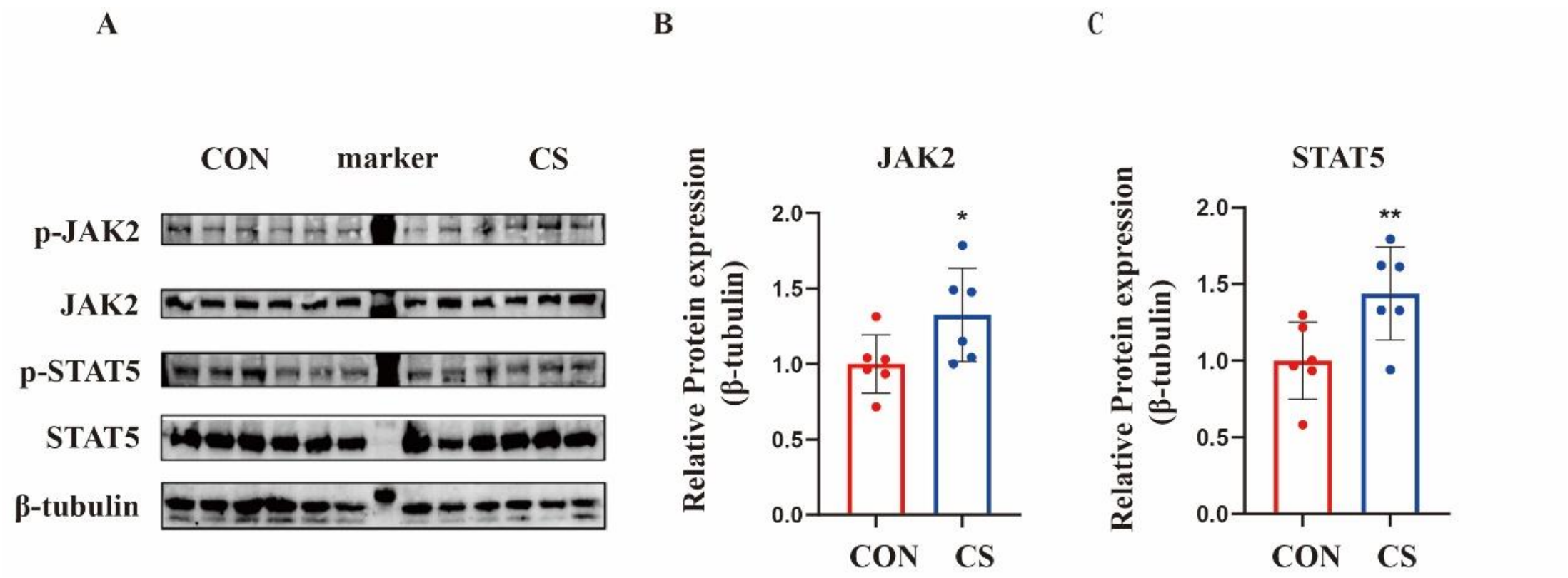

Supplement 5. Effects of cysteamine supplementation in diets on protein expression level of JAK2-STAT5 signaling pathway in *longissimus dorsi* muscle of *Hu* lambs. (A) The chemiluminescence of bands detected by a ChemDoc XRS+ imaging system. The relative protein expression of (B) JAK2, (C) STAT5 against the reference protein β-tubulin. Each group contained 6 *Hu* lambs fed either a CON or CS (cysteamine) diet. Values are the mean ± SEM. The red and blue represent the value of each sample, and the height of the bar represents the average value of each group. $*0.05 < p < 0.10$, $**p < 0.05$.
